# Oxidation differences on Si- versus C-terminated surfaces of SiC during planarization in the fabrication of high-power, high-frequency semiconductor device

**DOI:** 10.1038/s41598-023-49622-z

**Published:** 2023-12-21

**Authors:** Ganggyu Lee, Yeram Lee, Sungmin Kim, Donghwan Kim, Hongjun Park, Myungju Woo, Taeseup Song, Ungyu Paik

**Affiliations:** Department of Energy Engineering, Hanyang University, Seoul, Republic of Korea

**Keywords:** Engineering, Materials science, Nanoscience and technology

## Abstract

Silicon carbide (SiC) wafers have attracted attention as a material for advanced power semiconductor device applications due to their high bandgap and stability at high temperatures and voltages. However, the inherent chemical and mechanical stability of SiC poses significant challenges in the chemical mechanical planarization (CMP) process, an essential step in reducing defects and improving surface flatness. SiC exhibits different mechanical and chemical properties depending on SiC terminal faces, affecting SiC oxidation behavior during the CMP process. Here, we investigate the process of oxide layer formation during the CMP process and how it relates to the SiC terminal faces. The results show that under the same conditions, the C-terminated face (C-face) exhibits higher oxidation reaction kinetics than the Si-terminated face (Si-face), forming an oxide layer of finer particles. Due to the different oxidation kinetic tendencies, the oxide layer formed on the C-face has a higher friction coefficient and more defects than the oxide layer formed on the Si-face. This results in a higher removal rate during CMP for the C-face than the Si-face. Furthermore, by controlling the physicochemical properties of the oxide film, high removal rates can be achieved by friction with the pad alone, without the need for nanoparticle abrasives.

## Introduction

Silicon carbide (SiC) is a promising material in advanced power semiconductor devices due to its notable high bandgap (3.26 eV, The bandgap of conventional Si is 1.12 eV.) and remarkable resilience to high temperatures and voltages^1–3^. The unique physical and electrical properties of SiC allow it to handle high power density and enable operations at elevated frequencies with reduced energy loss, thus outperforming traditional materials like silicon^4^. These attributes are particularly beneficial in various demanding power electronics applications, from electric vehicles and renewable energy systems to high-power radio frequency (RF) device. SiC presents a variety of crystal structures, including 3C-SiC, 4H-SiC, and 6H-SiC. Among these, the 4H-SiC structure garners significant attention in power electronics. The preference for 4H-SiC results from its superior performance characteristics, such as excellent electron mobility and thermal conductivity, compared to other SiC crystal structures. Despite these superior properties, the difficulty in producing SiC wafers still limits their practical use^5^.

In particular, one of the most difficult processes is chemical mechanical planarization (CMP), which smooths the surface of a device to improve its optical and electrical properties. The CMP process combines mechanical polishing with chemical reactions to remove irregularities on the wafer surface, control the thickness of the wafer, and prepare the wafer surface for subsequent processes such as photolithography, pattern transfer, and interlayer insulator removal^6^. Due to high hardness and chemical stability (the same properties that make it desirable), the removal rate of 4H-SiC is too low during CMP, which makes it difficult to achieve the desired degree of wafer surface planarization. 4H-SiC has two different face orientations, Si-terminated ((0001), Si-faces) and C-terminated ((000-1), C-faces), which exhibit different mechanical and chemical properties. CMP, carried out to planarize their surfaces, is a continuous process in which an oxidizing agent forms a weak oxide layer and removes it with an abrasive. Therefore, to achieve a high removal rate, it is necessary to compare the effects of the oxide film formed during CMP on the surfaces of the Si-face and C-face^7^.

Numerous researchers have dedicated their efforts to engineering CMP slurry to achieve a high removal rate in SiC CMP. H.S. Lee et al. and X. Fu et al. reported that high-hardness abrasives, such as SiC, alumina, and nanodiamond improve the SiC CMP removal rate^8,9^. However, using such high-hardness abrasives can lead to surface defects and scratches on the SiC material, posing a challenge. To address this issue, Lu et al. and Zhou et al. have explored the effectiveness of polishing SiC after oxidizing the surface to form an oxide layer, enabling high removal rates^10–12^. They have highlighted the formation of an oxide layer on the SiC surface through oxidizers, followed by CMP with a low-hardness abrasive like silica. Among the oxidizers studied, U. Lagudu et al. has found that KMnO_4_ exhibits the highest oxidizing effect. Additionally, S. Babu et al. has reported promoting SiC oxidation in the acidic region, where the reduction reactivity of KMnO_4_ is high.

Previous studies on SiC CMP have shown that high removal rates can be achieved by forming an oxide layer on SiC. However, a comprehensive review of these studies led us to recognize the need for further research into the physical and chemical properties of the oxide layer and a deeper understanding of how the different face orientations of 4H-SiC behave differently during the oxidation process. In this study, we focused on investigating the formation of oxide layers and their effects on CMP performance. We aimed to determine which microstructures and compositions of oxide layers favor CMP. As previously reported, we compared and investigated the formation of the oxide layer on the SiC surface in the different face orientations (Si-face and C-face) using CMP slurry titrated to pH 2.5 with KMnO_4_ oxidizer, and studied how the characteristics of the oxide layer formed at different face orientations affect CMP.

Here, we analyze the chemical composition of the oxide layer formed for each face orientation during the CMP process and evaluate its mechanical properties, including layer thickness, friction, and hardness. The results show that the oxidation reaction kinetics of the C surface is higher than that of the Si surface under the same conditions. The higher oxidation reaction kinetics leads to the formation of more SiO_2_ compared to the SiC_x_O_y_ ratio, which in turn leads to the escape of CO or CO_2_, forming an oxide layer composed of fine particles. The fine microstructure of the oxide layer results in lower hardness due to defects at the intergranular boundaries and a higher coefficient of friction at the intergranular boundaries, resulting in a higher removal rate. Furthermore, controlling the physicochemical properties of the oxide layer allows high removal rates to be achieved by friction with the pad alone, without the need for nanoparticle abrasives.

## Results and discussion

### Oxidation mechanism and oxide layer formation on Si-face and C-face SiC wafers in CMP slurry

**Figure 1:**
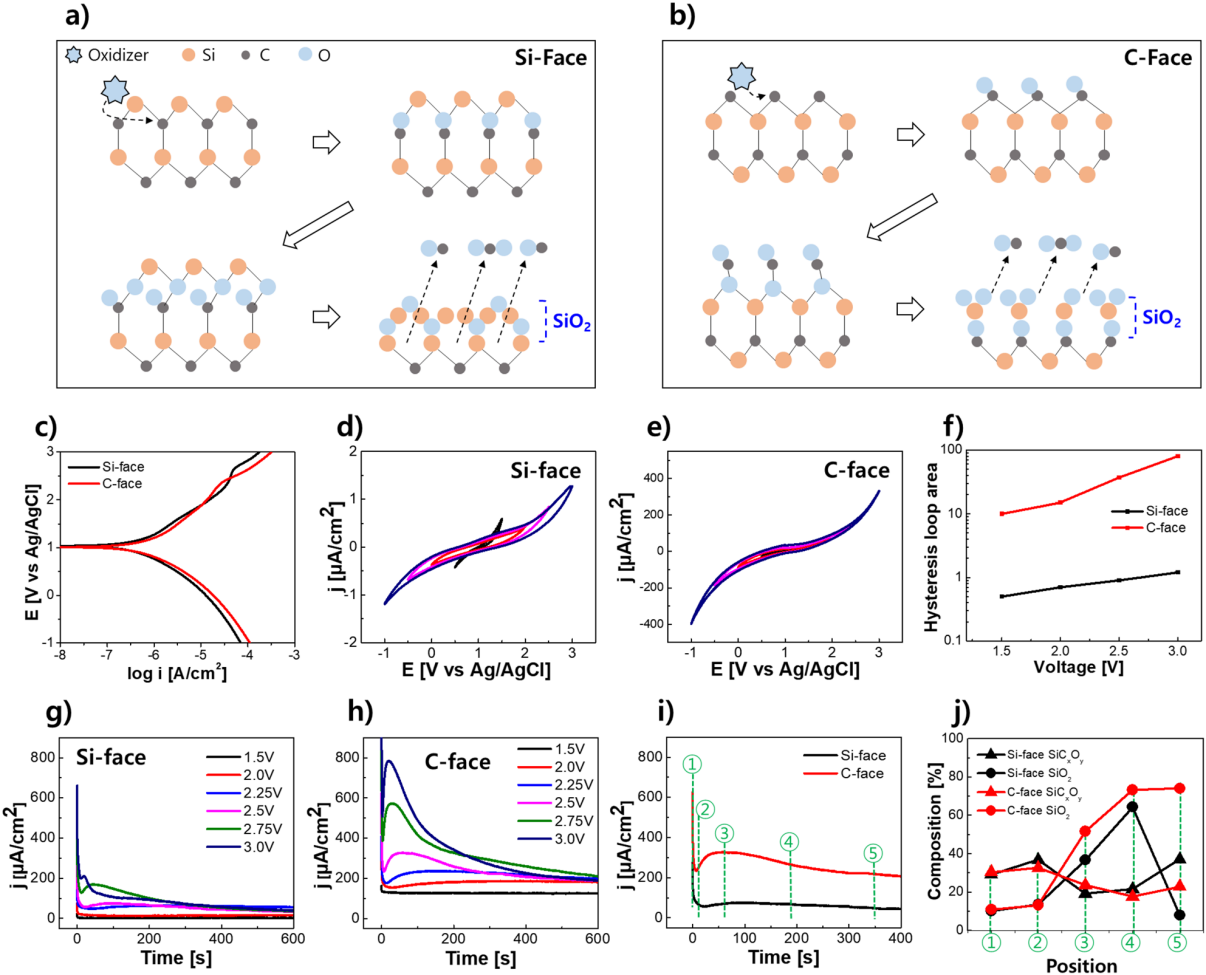
Schematic illustration of the oxidation mechanism by an oxidizer on the (**a**) Si-face and (**b**) C-face of SiC. (**c**) Tafel plots comparing Si-face and C-face. (**d**) and (**e**) Cyclic voltammetry (CV) results with Ag/AgCl reference electrode on Si-face and C-face, respectively. (**f**) Hysteresis loop area (V) obtained from CV measurements. (**g**) and (**h**) Chronoamperometry results on Si-face and C-face, respectively. (**i**) Current density over time for each orientation direction at a voltage of 2.5 V. (**j**) Composition of the oxide layer at positions ① to ⑤ in (**i**).

Figure 1a and b illustrate the oxidation mechanism on the Si-face and C-face of SiC. When an oxidizer attacks SiC, an oxidation reaction takes place. In this process, the oxidizer targets the C atom, which has a higher electronegativity. As a result, an intermediate compound called SiC_x_O_y_ is formed on the surface of the oxidized SiC. Eventually, the C atoms are released as CO or CO_2_, while SiO_2_ is produced. When comparing Si-face and C-face under the same oxidizer, C-face outperforms Si-face regarding a structural steric hindrance when attacked by an oxidizer and when CO or CO_2_ escapes to the final product^13^.

Figure 1c shows Tafel plots of Si-face and C-face SiC wafers in CMP slurry. Both slurry samples exhibit a corrosion potential of 1.08 V (Ag/AgCl reference electrode), indicating consistent thermodynamic characteristics of the corrosion reaction. The corrosion potential represents the electrochemical potential between the oxidizer and SiC in the slurry, reflecting the balance between reduction and oxidation reactions in the system^14^. It serves as an indicator for evaluating the corrosion propensity of the material. The similarity in corrosion potential for each face direction implies that both surfaces have similar thermodynamic tendencies for oxidation reactions. However, there is a discrepancy in the corrosion current. Corrosion current represents the reaction kinetics and signifies the rate of the corrosion reaction^15^. As observed in the Tafel plot graph, the Si-face exhibits a corrosion current of 0.51uA/cm^2^, while the C-face exhibits a corrosion current of 0.73uA/cm^2^. The difference in corrosion current between Si-face and C-face indicates that the oxidation reaction rate is faster on the C-face than on the Si-face.

To investigate how the difference in oxidation reaction rate, based on the direction of the crystal plane, affects the formation of oxide films, cyclic voltammetry (CV) was performed. CV measurements were carried out considering the previously observed corrosion potential of 1.08 V (Fig. 1d and e). In the CV data, a hysteresis loop refers to a phenomenon where the forward and backward scan curves do not perfectly overlap, resulting in a closed loop shape^16–18^. This occurrence arises due to differences in the rate constants of the forward and reverse reactions, resulting in the stable oxide film formed by the anodic reaction on the SiC surface not wholly disappearing during the cathodic reaction. The area of the hysteresis loop serves as an indicator of the extent of oxide film formation. As depicted in Figs. 1f, the hysteresis loop area in the C-face is approximately 200 times larger than that of the Si-face. That means demonstrating that oxide film forms faster and, to a greater extent, on the C-face.

Based on the CV data, current voltammograms (current density vs. time) were obtained using chronoamperometry in the voltage range of 1.5–3.0 V (Fig. 1g and h). The voltammograms display the variation of surface current over time as the oxidation reaction progresses with changing potential. Measurements were conducted for each surface direction. Initially, when a potential is applied, polarization occurs, and the current rapidly drops. Subsequently, the current density increases due to charge transfer through the redox reaction, forming an oxide film on the surface. The peak current density appears around 20 to 50 s, with the C-face exhibiting a more significant peak of higher anodic current, indicating a lower activation barrier for the oxidation reaction. Over time, the current density gradually decreases and reaches a saturation point. This decrease is attributed to the surface oxide film acting as a passivation layer, hindering further reaction between SiC and the oxidizer and lowering the reaction kinetics.

In the case of the Si-face, the peak starts to appear at 2.5 V. At this voltage, a current density of 74.76 uA/cm^2^ is observed after 100 s, followed by a gradual decrease in current density. At 2.75 V, a current density of 166uA/cm^2^ is recorded after 58 s, and at 3 V, a peak of 209uA/cm^2^ is observed after 20.7 s, followed by a decrease in current density. On the other hand, in the C-face, at 2.5 V, a peak of 325uA/cm^2^ is observed at 62.8 s. At 2.75 V, a current density of 571 uA/cm^2^ appears after 27.13 s, and at 3 V, a peak of 781.6uA/cm^2^ is observed at 23.4 s. The current density generated during the initial oxide layer formation on the C-face is higher than that on the Si-face at all voltages, and the time taken for the oxide film to form is faster. This suggests that the C-face exhibits faster kinetics for the initial oxide film formation compared to the Si-face, consistent with the previous Tafel plot and CV data findings.

Figure 1i represents the current overtime for Si-face and C-face when a voltage of 2.5 V is applied. Figure 1j shows the compositional analysis of the SiC oxide film by dividing the graph into five sections where the shape changes. Initially (①), the ratio of SiO_2_ and SiC_x_O_y_ in both Si-face and C-face is almost identical. After polarization, there is no significant change in composition (②). However, once the oxide film forms due to the initial oxidation reaction, the composition of the Si-face and C-face starts to diverge. SiC_x_O_y_ formed on the initial surface in both faces further oxidizes to form SiO_2_, observed in sections ③ and ④. However, in the final ⑤ sections, there is a difference. While the C-face exhibits a higher SiO_2_ ratio, indicating the progress towards SiO_2_ formation, the Si-face shows a higher SiC_x_O_y_ ratio, suggesting that the reaction does not proceed to SiO_2_ but only to the SiC_x_O_y_ form. This is likely because, as illustrated in Fig. 1a, the reaction does not reach the final stage due to the inability of the C atom in the form of CO and CO_2_ to escape.

### Composition and structural difference of oxide layer on Si-face and C-face SiC wafers in CMP slurry

**Figure 2:**
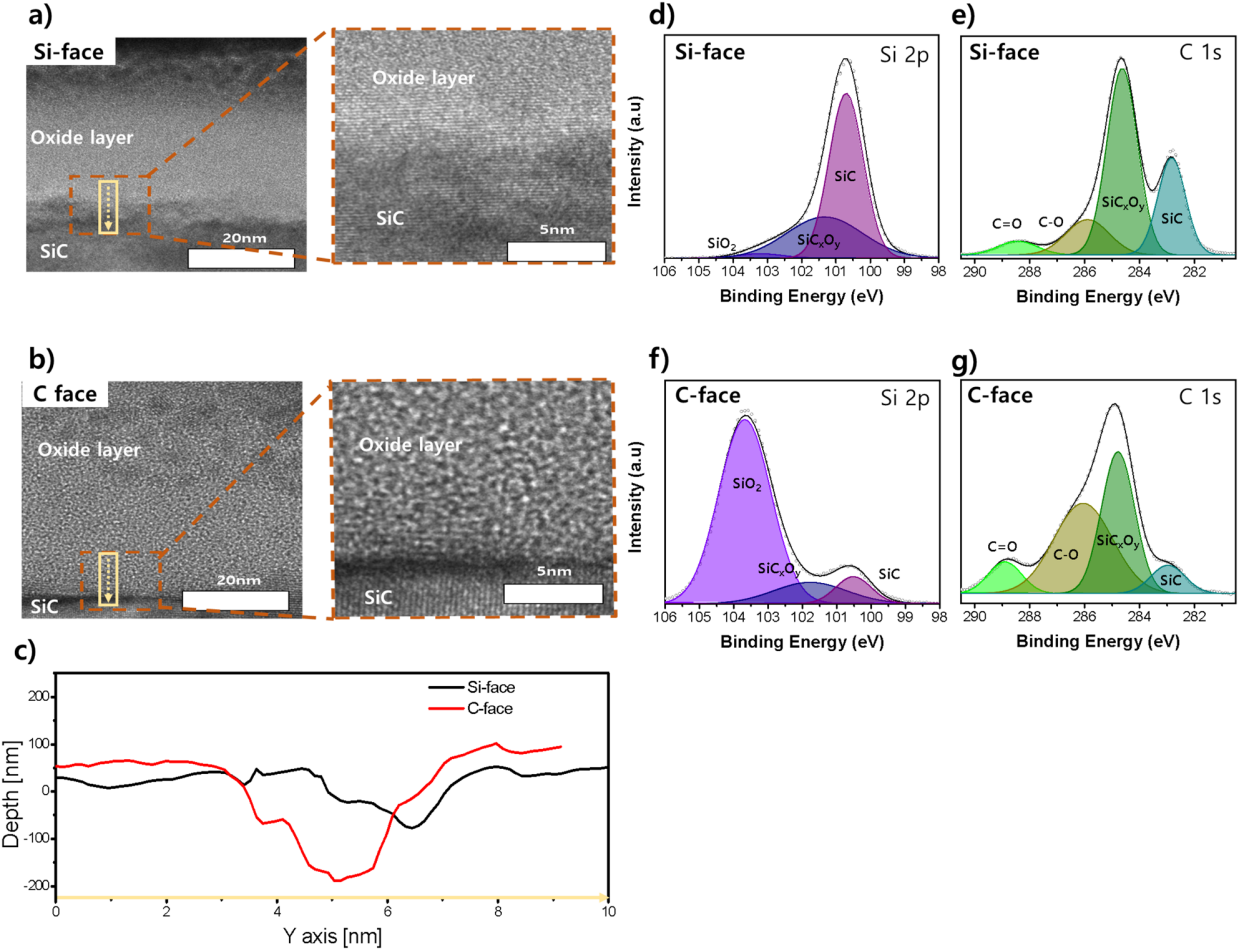
(**a**, **b**) TEM and high-resolution TEM measurement results of the interface between SiC and oxide layers on the Si-face and C-face, respectively. (**c**) Line profile of the yellow boxed region in the TEM data measured by AFM. (**d**, **f**) XPS measurement results of the oxide film on the Si-face and C-face show the Si 2p peak. (**e**, **g**) XPS measurement results of the oxide film on Si-face and C-face, respectively, showing the C 1 s peak.

The structure and composition of the oxide layer formed on each crystal plane were analyzed. TEM measurements in Fig. 2a and b reveal the interface between the oxide layer and SiC after immersion in CMP slurry. The oxide layer on the Si-face was approximately 25 nm thick, while it measured about 39 nm on the C-face. The thicker oxide layer on the C-face can be attributed to the higher corrosion current and constant voltage current observed during electrochemical evaluation, indicating increased oxidation reactions. High-resolution TEM analysis identified defects at the interface between the SiC and oxide layers for both the Si-face and C-face. However, whereas the defects on the Si-face appeared scattered and separated, continuous defects were observed on the C-face.

Further characterization through atomic force microscopy (AFM) line profile measurement, as shown in Fig. 2c, provided a clearer view of the identified defects within the yellow boxed areas in Fig. 2a and b. The dark regions observed in the TEM image correspond to deep valleys observed in the AFM line profile, with depths measuring 30 nm on the Si-face and 220 nm on the C-face (Fig. 2c). These observations indicate that the dark areas in the TEM image are line defects between the SiC and the oxide layer formed on its surface.

The composition of each oxide layer was determined through X-ray photoelectron spectroscopy (XPS) analysis of the Si 2*p* and C 1*s* peaks. Figure 2d and e show the Si 2*p* and C 1*s* peaks of the oxide layer formed on the Si-face, respectively. The Si 2*p* peaks at 100.8 eV, 101.5 eV, and 103.3 eV represent SiC, SiC_x_O_y_, and SiO_2_, respectively^19^. The relative peak areas show that the oxide layer consists of approximately 62.83% SiC, 37.3% SiC_x_O_y_, and 8% SiO_2_. The results from the C 1*s* peak analysis align with those of the Si 2*p* analysis. The C 1*s* peaks at 283.0 eV, 284.8 eV, 286.6 eV, and 288.6 eV correspond to SiC, SiC_x_O_y_, C–O, and C=O bonds, respectively^20^. The experimental results indicate peak areas of approximately 25.9% SiC, 54.6% SiC_x_O_y_, 14.4% C–O, and 5% C=O.

On the C-face, the analysis of the Si 2*p* peak reveals that the oxide layer consists of approximately 7.8% SiC, 12.7% SiC_x_O_y_, and 79.4% SiO_2_ (Fig. 2f). This higher proportion of SiC on the C-face indicates the formation of a thicker oxide layer, which also contains a higher percentage of SiO_2_ than SiC_x_O_y_. The C 1*s* peak analysis results support these findings, showing approximately 8.1% SiC, 39.1% SiC_x_O_y_, 44.1% C–O, and 8.8% C=O (Fig. 2g). The ratio of C-O and C=O, representing the final oxidation forms of the C atom, is approximately 52.9%, about 2.7 times higher than that observed on the Si-face (19.5%).

The rapid progression of oxidation on the C-face leads to modifications in the microstructure of the oxide surface due to the release of C atoms as by-products. This process creates numerous defects that facilitate the penetration of the oxidizer and the smooth emission of CO or CO_2_. In other words, the initial formation of the oxide layer occurs quickly on the C-face due to its structural advantage with less steric hindrance. However, subsequent oxidation reactions proceed at an accelerated rate due to the easy attack and removal of by-products by oxidizers through the existing defects. This demonstrates the enhanced oxidation promotion on the C-face compared to the Si-face.

### Investigating the mechanical properties of oxide films on Si-face and C-face of SiC

**Figure 3:**
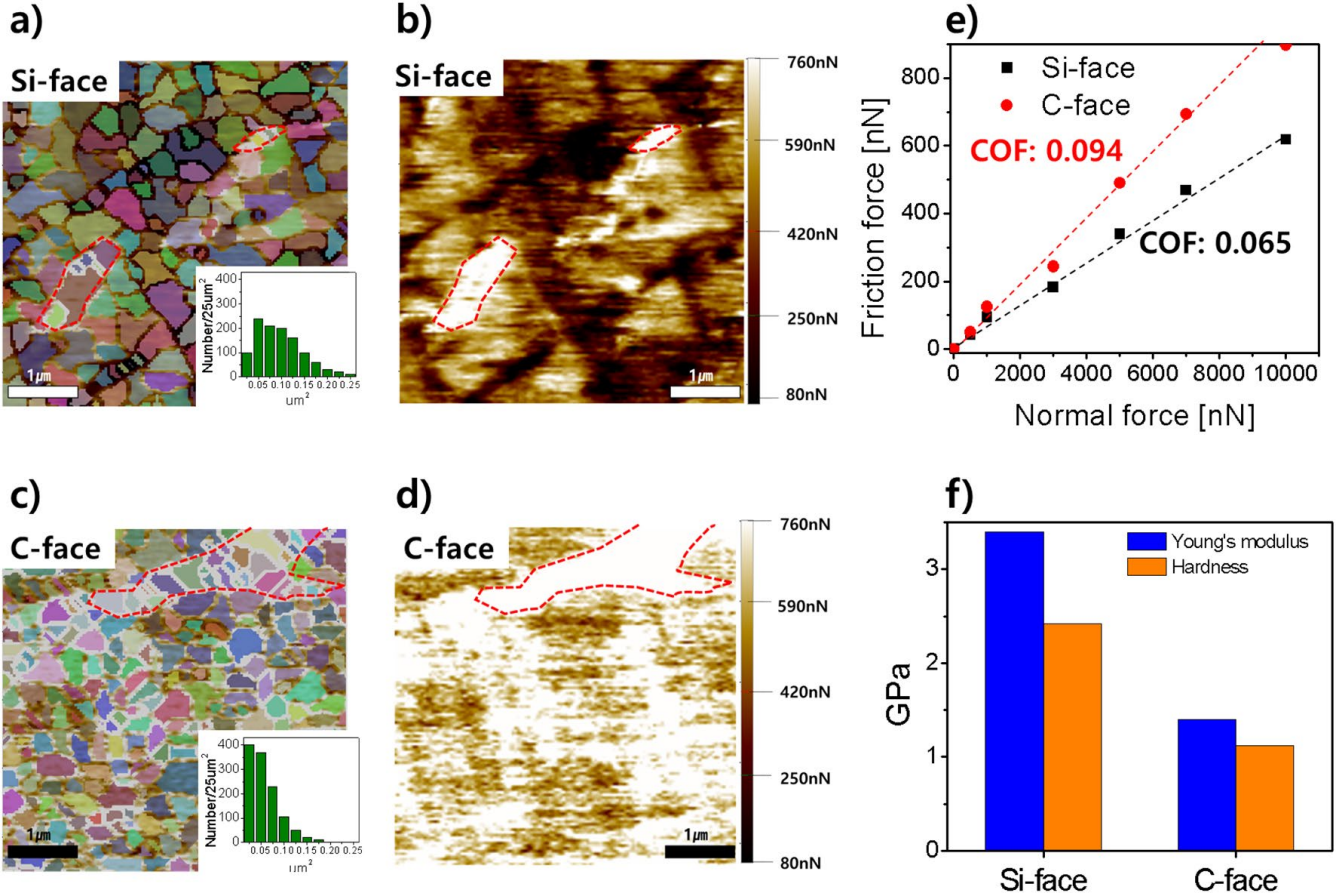
(**a**, **c**) AFM measurements show the topography and fine particle boundary of the Si-face and C-face, respectively. (**b**, **d**) Lateral force (friction force) measurements on Si-face and C-face. (**e**) Coefficient of friction (COF) of the oxide layer for each orientation direction of SiC. (**f**) Young's modulus and hardness measurements of the oxide layer in each orientation direction of SiC.

Figure 3a and c depict the AFM measurements of the oxide layer's fine particles and their boundaries on the Si-face and C-face of SiC. In the Si-face, 912 fine particles were observed within a 25 μm^2^ area, while the C-face exhibited 1,052 fine particles. Additionally, the particle size analysis revealed that the particles formed on the C-face were primarily composed of smaller particles than those observed on the Si-face. Figure 3b and d represent the surface lateral forces of oxidized Si-faces and C-faces in the same slurry, measured when the AFM tip is moved horizontally with the same normal force (10 μN). In this case, the lateral force refers to the frictional force, with higher lateral forces indicating higher frictional forces. The average friction forces on the Si-face and C-face are 618 nN and 897 nN, respectively. The C-face friction force is about 45% higher than the Si-face. What is noticeable is that the regions that exhibit higher lateral forces (regions colored white and red in Fig. 3b and d) are mainly associated with the boundaries of the fine particles observed in Fig. 3a and b. In other words, the points with higher friction forces correspond to the boundaries of the fine particles, and the C-face experiences higher friction forces under the same normal force compared to the Si-plane due to the presence of more such boundaries. Furthermore, a graph showing the average value of the friction force under normal force is shown in Fig. 3e, where the Si-face and C-face exhibit values of 0.065 and 0.094, respectively. This indicates that the fine particle boundaries in the oxide layer cause high frictional forces, and is the reason that the C-face, which has a relatively larger number of boundaries, has higher frictional forces than the Si-face.

Interestingly, when measuring the friction force of the polyurethane-based CMP pad using the same method, a value of 0.12 was obtained. Conventionally, CMP processes involve the combined action of chemical additives reacting with the CMP slurry surface and the mechanical effect of nanoparticle abrasives for film removal. Silica, ceria, and alumina are commonly used as nanoparticle abrasives, with silica nanoparticles being the most prevalent choice to minimize wafer surface scratches. However, aggregation of silica nanoparticles in the slurry can lead to issues such as within-wafer non-uniformity and scratches. While CMP without abrasives offers advantages, its practical application is challenging due to the low removal rate. Nonetheless, the current experimental results demonstrate that an abrasive-free CMP pad can achieve a sufficiently high frictional force on both the Si-face and C-face. This indicates the possibility of abrasive-free CMP by controlling oxide layer defects, thickness, and composition in SiC CMP, providing hope for future applications.

Figure 3f shows the oxide film's mechanical strength measurements on each crystal plane. The Si-face, with fewer defects and larger surface oxide particles, exhibited Young's modulus of 3.4 GPa and a hardness of 2.42 GPa. In contrast, the C-face displayed a lower Young's modulus of 1.4 GPa and a hardness of 1.12 GPa. When comparing these values to those of the CMP pad, which had a hardness of 1.7 GPa, it suggests that the oxide film formed on the C-face, with relatively lower mechanical strength, can be effectively removed using the CMP pad alone.

### CMP evaluation results of SiC for each orientation direction

**Figure 4:**
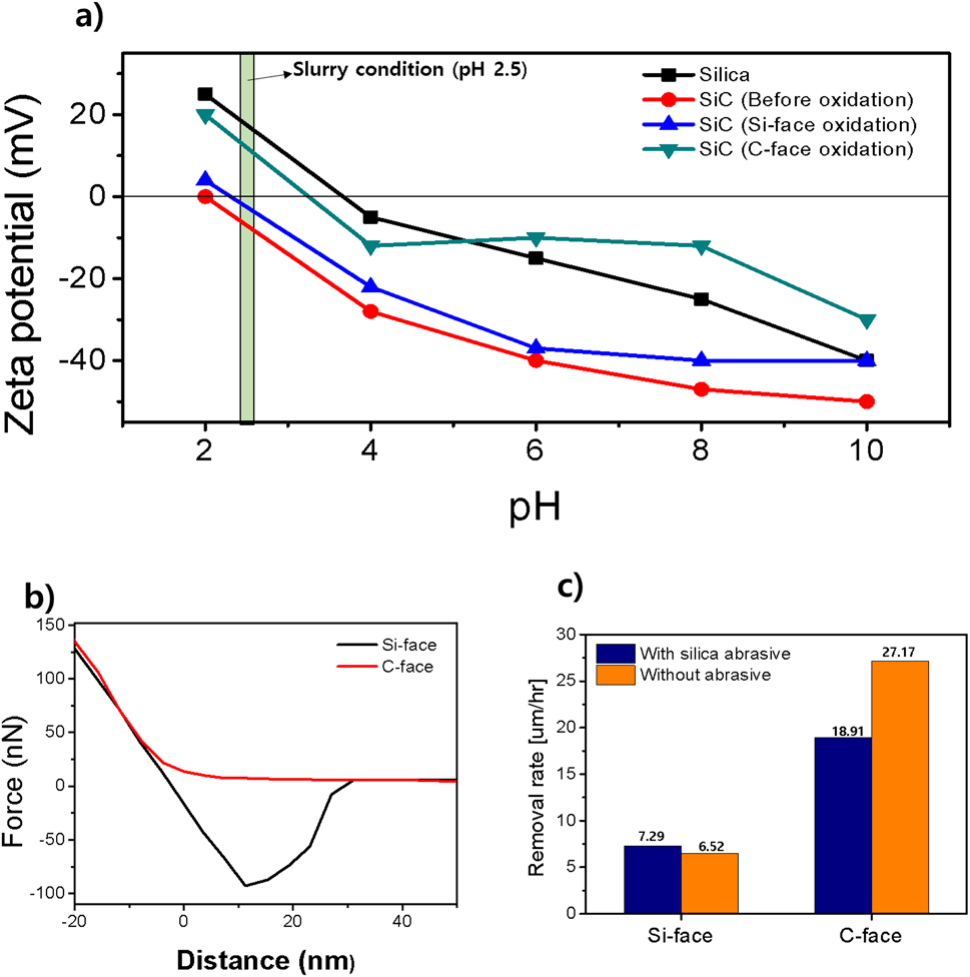
(**a**) Zeta potential measurements of silica (abrasive), pre-oxidized SiC, a Si-face oxide layer, and a C-face oxide layer at different pH values. (**b**) Force-distance curve (F-D curve) at pH 2.5 between each oxide layer and silica. (**c**) CMP results for each plane direction with and without silica abrasive.

Figure 4 demonstrates the CMP process in different crystal plane directions, both with and without silica nanoparticle abrasives. Figure 4a shows the zeta potential of silica and SiC and the pre-oxidized SiC wafers, representing each plane direction. Despite the same oxidation conditions (which means that the slurry conditions for pre-oxidation of the SiC wafers were the same), differences in the kinetics of the Si-face and C-face oxidation reactions resulted in different thicknesses of the oxide layers in each plane direction. The difference in isoelectric point (IEP) shift is chiefly due to that of oxide thickness. The results indicate an IEP of 3.8 for silica and an IEP of 2.1 for SiC. Regarding the oxidized SiC surfaces, the IEP of the oxide layer formed on the Si-face was 2.3, while the IEP of the oxide layer formed on the C-face was 3.6. The C-face, characterized by a thicker oxide layer, exhibits a zeta potential closer to that of silica, while the Si-face, with a thinner oxide layer, aligns with the zeta potential of SiC. The CMP slurry used in the experiments was adjusted to a pH of 2.5. Consequently, silica abrasives generate electrostatic attractive forces on the Si-face, while silica and electrostatic repulsive forces are predominantly observed on the C-face.

This can be further confirmed by examining the force-distance (F-D) curve at pH 2.5. Analysis of the force between the silica probe and the oxide layer for each plane direction reveals an attractive force on the Si-face starting from 7 nm, while a repulsive force is observed on the C-face starting from 28 nm (Fig. 4b). This indicates that silica abrasives have better access to the oxide layer on the Si-face, suggesting a potential improvement in the removal rate.

The CMP results substantiate this phenomenon (Fig. 4c). The Si-face exhibits a removal rate of 7.29 μm/h with silica abrasives, compared to 6.52 μm/h without abrasives. In contrast, the C-face demonstrates a removal rate of 18.91 μm/h with abrasives, which significantly increases to 27.17 μm/h without abrasives. The higher removal rate on the C-face can be attributed to the mechanical effects of the oxide layer. Notably, the higher removal rate without abrasives suggests that under the pressurized conditions typically employed in CMP (6 psi), the mechanical effects of silica abrasives are hindered, thereby suppressing the chemical reaction. Importantly, these findings reveal that a sufficiently high removal rate can be achieved without using abrasives in the CMP slurry, where the Si-face and the C-face represent oxide layers with numerous defects.

## Conclusion

This study enhances our understanding of the CMP process for preparing SiC wafers. Electrochemical experiments have revealed the significant influence of SiC crystal face orientation on oxide film formation during CMP. The kinetics of oxide film formation plays a crucial role, with higher kinetics leading to increased SiO_2_ formation compared to SiC_x_O_y_, producing fine-grained oxide films. These oxide layers exhibit a high coefficient of friction, leading to enhanced removal rates during CMP.

The findings of this study have important implications for the fabrication of advanced power semiconductor devices using SiC wafers. Firstly, understanding the role of crystal plane orientation and oxide film formation kinetics can enable more effective and efficient SiC wafer preparation. Secondly, the study suggests the possibility of achieving high removal rates by manipulating the physicochemical properties of the oxide layer, potentially eliminating the need for nanoparticle abrasives in the CMP process. This novel approach can simplify the CMP process, improving manufacturing efficiency and reducing costs.

Furthermore, this study opens avenues for future research. Exploring the impact of factors such as temperature, pressure, pH, and slurry chemistry (e.g., oxidizers and catalysts) on the oxidation reaction kinetics of SiC and the efficiency of the CMP process could optimize CMP conditions. In summary, these findings provide a foundation for developing more efficient, cost-effective, and streamlined processes for SiC wafer preparation, highlighting the potential of SiC in advanced power semiconductor device applications.

## Experimental section

### Materials preparation

Commercially colloidal SiO_2_ was used as an abrasive (dmean ~ 100 nm, Fuso, Japan). The solid concentration of SiO_2_ was 5.0 wt%. 3.0 M potassium permanganate (KMnO_4_, Sigma Aldrich, USA) was used as an oxidizer. The concentration of ferric (III) nitrate nonahydrate (Sigma Aldrich, USA) as a catalyst was 4 mM. The slurry pH was adjusted to 2.5 using nitric acid (HNO_3_, 1.0 N, Daejung Chemical, Korea). We utilized 6-inch commercial 4H-SiC single crystal wafers from SICC Co., Ltd., which were characterized by nitrogen doping at a concentration of 10^17^ cm^−3^ and a resistivity of 0.021 Ω cm. The wafers have an original thickness of 350 (± 25) μm and had been preliminarily polished by double sides lapping. The processed wafer has a high degree of planar and parallel on the two sides. Due to the crystal structure of 4H-SiC wafers, the Si-face and C-face always appear as a pair: if one side of the wafer is Si-face, the other is always C-face. All experiments with the wafers were performed by dipping them in a 150:1 buffered oxide etchant solution for 15 min to remove native oxide before the experiment.

### Oxidation behavior and oxide layer properties analysis

The oxidation behaviors of the SiC according to face orientation were analyzed through X-ray photoelectron spectroscopy (XPS) (K-Alpha+, Thermo Fisher Scientific Messtechnik, USA). The SiC films were dipped in 200 mL at pH 2.5 for 15 min. Then samples were subsequently rinsed with deionized water before analysis. Morphological characteristics were conducted using a high-resolution transmission electron microscope (HRTEM, JEOL 2010).

To investigate whether electrostatic attraction or repulsion exists between the oxidized SiC and SiO_2_ abrasive surfaces during the CMP process, we measured the zeta potential as a function of pH of the SiO_2_, SiC, and SiC oxidized in each face direction (Si-face and C-face). The zeta potential values of SiC and SiO_2_ correspond to the pre-oxidized SiC film and abrasive nanoparticles, respectively. These values were measured using a Nano ZS instrument (Malvern Instruments Ltd.) by electrophoresis in suspension. Oxide layers were formed for each orientation of SiC, and their zeta potentials were determined using streaming potential measurements performed with a tunable gap cell in a SurPASS 3 system (Anton Paar). We measured the induced streaming potential by stacking identical films face to face and flowing a solution pH-adjusted with HNO_3_ and KOH between them.

Force-distance curves and lateral forces were performed with an AFM (XE-150, Parksystems) in contact and lateral force measurement mode with a scan size of 5 × 5 μm and a scan rate of 0.5 Hz using a Si cantilever with a SiO_2_ tip. In contact mode, the vertical bending of the cantilever is monitored while the AFM tip scans the surface to gather information about the surface morphology of the sample, in lateral force mode, a constant force is set in the vertical direction and then the torsion of the cantilever is monitored as the tip moves in the horizontal direction to gather information about the distribution of friction properties on the sample surface. If the force acting in a horizontal direction is large (high friction force), the cantilever twists more from side to side. Coefficient of friction (COF) was obtained by measuring the lateral force (friction force) at 0, 500, 1,000, 3,000, 5,000, 7,000, and 10,000 nN for normal force and the slope of normal force and friction force.

### Electrochemical performance investigation

Potentiodynamic polarization measurement, cyclic voltammetry, and chronoamperometry measurement (AUT320N, Metrohm AUTOLAB, Switzerland) were used to characterize the electrochemical performance of SiC. The counter electrode was a platinum-coated mesh, and the reference electrode was an Ag/AgCl containing 3 M KCl electrolyte. The size of cut samples for electrochemical measurements were 1 × 3 cm^2^ (exposing 1 cm^2^ active area), respectively. Before each experiment, the SiC wafer was removed from the native oxide using a buffered oxide etch (BOE) solution (Sigma-Aldrich, USA).

### CMP performance evaluation

The CMP evaluation to obtain the material removal rate of SiC was performed for 20 min at 6.0 psi pressure, 197/200 rpm rotational speed of the head/platen, respectively, and 200 mL/min flow rate. The change in mass after CMP was measured to determine the material removal rate.

## Data Availability

All data generated or analyzed during this study are included in this published article.
